# Characterization of white blood cell ratios in South American camelids presented at a veterinary teaching hospital

**DOI:** 10.1038/s41598-024-76985-8

**Published:** 2024-10-29

**Authors:** Matthias Gerhard Wagener, Max Kornblum, Martin Ganter, Frederik Kiene

**Affiliations:** Clinic for Swine, Forensic Medicine and Ambulatory Service, Small Ruminants, University of Veterinary Medicine Hannover, Foundation, Hannover, Germany

**Keywords:** Alpaca, Llama, Clinical pathology, White blood cells, Diagnostic marker, Stress, Animal physiology, Diagnostic markers

## Abstract

White blood cell (WBC) ratios are used as diagnostic markers for various inflammatory or tumor diseases as well as stress in a broad range of species. The aim of this work was to provide data on five WBC ratios (neutrophil-to-lymphocyte ratio [NLR], band neutrophil-to-lymphocyte ratio [BLR], band neutrophil-to-neutrophil-to-lymphocyte ratio [BNLR], band neutrophil-to-neutrophil ratio [BNR] and lymphocyte-to-monocyte ratio [LMR]) in South American camelids (SAC) and characterize their association with demographic and important diagnostic parameters. Medical records of 307 SAC (275 alpacas, 32 llamas) that were presented at a veterinary teaching hospital were evaluated retrospectively. WBC ratios were calculated based on hematologic results of the initial blood samples. The influence of species, sex, age, body condition score, WBC count, and anemia on those ratios was investigated using descriptive statistics and generalized linear models. NLR, BLR and LMR were found to be significantly influenced by age and WBC count. Associations of individual WBC ratios with species, nutritional status or an anemic condition could be detected. NLR was 4.32; 2.31–7.81 (median; IQR), BLR 0.24; 0.07–0.87, BNLR 3.66 × 10^–3^; 1.17 × 10^–3^ − 14.20 × 10^–3^, BNR 0.06; 0.02–0.15 and LMR was 7; 3.54–14.67. Our data might serve as a basis for further studies on WBC ratios in SAC. The animals in this study showed a variety of underlying diseases. It should hence be noted that these values are orientation values and provide a representative overview of conditions in a clinic, but are not suitable as reference values for healthy animals.

**Supplementary Information:**

The online version contains supplementary material available at 10.1038/s41598-024-76985-8.

## Introduction

Clinical pathology plays an important role in the veterinary care of South American camelids (SAC, alpacas and llamas). Due to the stoic character of these animals, clinical symptoms of disease like emaciation and anemia often remain undetected for a long time^1–3^. A large number of the animals presented at the clinic exhibit a reduced Body Condition Score (BCS) and a decreased packed cell volume (PCV), which is usually closely associated with each other^2^. The BCS is not only associated with the red blood count, but also with the WBC (white blood cell) differential. In llamas, we observed increased neutrophils and decreased lymphocytes in animals with low BCS^2^. Based on conventional WBC differential data, further hematologic parameters can easily be calculated: the neutrophil-to-lymphocyte ratio (NLR), which is used as a prognostic marker for tumor diseases^4^, or inflammatory conditions such as chronic enteropathies^5^ or mastitis^6^ in other species. The neutrophil-to-lymphocyte ratio was already mentioned for both alpacas^7–9^ and llamas^10–12^. Further information on the characteristics of these parameters is, however, lacking in those publications, which are limited to reference intervals of a few animals or individual case reports. Nonetheless, NLR may also be of particular interest for SAC, as it has also potential not to be used only as a marker for disease, but also as a stress marker, which was already studied in other species^13–16^. Many factors like climate or social isolation can lead to stress in SAC^17,18^. Although there is little data on SAC compared to other species, stress is assumed to be a factor contributing to the development of gastric ulcers, which are commonly observed in camelid medicine^19,20^.

In addition to the NLR, other WBC ratios like lymphocyte-to-monocyte ratio (LMR), platelet-to-lymphocyte ratio (PLR), band neutrophil-to-lymphocyte ratio (BLR), or band neutrophil-to-neutrophil-to-lymphocyte ratio (BNLR) have been reported as prognostic markers for different inflammatory or tumor diseases in veterinary medicine^21–24^. However, to date, information on these parameters for SAC is completely absent in the literature.

In the present retrospective study, we therefore characterized five different WBC ratios in 307 SACs presented at our clinic. In addition to the ratios described so far (NLR, LMR, BLR, BNLR), we added the ratio of band neutrophils-to-neutrophils (BNR), which to our knowledge has not been described in the literature but may indicate the age progression of circulating neutrophils independently of lymphocytes. A higher proportion of young neutrophils (band neutrophils, metamyelocytes, myelocytes), preliminary stages of segmented neutrophils^25^, would increase the BNR.

Possible influences of species, sex, age, body condition score, WBC count, and anemia on each of those leukocyte ratios were tested using generalized linear models.

## Methods

### Data collection

For the retrospective data analysis, the findings of a total of 307 SACs were compiled from the electronic patient files and from the laboratory information system of the Clinic for Swine and Small Ruminants, Forensic Medicine and Ambulatory Service, University of Veterinary Medicine Hannover, Foundation, Hannover, Germany. The study was approved by the Ethics Committee of the University of Veterinary Medicine Hannover (approval code: TiHo-REC_14_10_24), all methods were performed in accordance with the relevant guidelines and regulations.

Data were collected from 275 alpacas (median age: 1586 days; IQR: 546–2636 days) and 32 llamas (median age: 1731 days; IQR: 704–3917 days) that were presented at the clinic between May 2019 and January 2023. As part of the clinical routine, a standardized clinical examination was carried out on admission of the animals, and a blood sample (EDTA Monovette 9 mL K3E, Sarstedt AG & Co. KG, Nümbrecht, Germany) was taken from the jugular vein of the animal and processed in the clinic’s own laboratory. Details of the hematologic examination were already described in a previous study^2^.

The following data were used in this study:

*Clinical data*

- Species: alpaca (A) or llama (L).
- Sex: male (m) or female (f).
- Age [days]: the age was calculated by subtracting the date of birth from the date of examination.
- Season: the month of admission of the animal to the clinic.
- BCS: BCS was determined by palpation of the lumbar spine and scored in 0.5 increments on a scale from 1 (emaciated) to 5 (obese)^26^.

*Hematologic data*

- PCV [l/l]: PCV was determined by centrifugation in microhematocrit capillary^2^.
- WBC count [10^9^/l]: WBC count was determined microscopically using a Neubauer counting chamber; if normoblasts (nucleated red blood cells) were found, the WBC count was corrected mathematically^2^.
- WBC ratios [dimensionless parameters]: WBC ratios were calculated from the results of the microscopic WBC differentiation. Therefore, 200 WBC from each stained blood smear were differentiated by trained laboratory personnel^2^.
  - NLR = (segmented neutrophils [%] + band neutrophils [%] + metamyelocytes [%] + myelocytes [%]) / lymphocytes [%].
  - BLR = (band neutrophils [%] + metamyelocytes [%] + myelocytes [%]) / lymphocytes [%].
  - BNLR = [(band neutrophils [%] + metamyelocytes [%] + myelocytes [%]) / segmented neutrophils [%]] / lymphocytes [%].
  - BNR = (band neutrophils [%] + metamyelocytes [%] + myelocytes [%]) / (segmented neutrophils [%] + band neutrophils [%] + metamyelocytes [%] + myelocytes [%])

Further categorical data were also created from some of the clinical and laboratory diagnostic findings:

- BCS-category: the animals were divided into two groups according to their nutritional status: lean-yes (BCS < 3) and lean-no (BCS ≥ 3).
- Anemia-category: the animals were divided into two groups according to their PCV: anemia-yes (PCV ≤ 25 L/L) and anemia-no (PCV > 25 L/L).
- WBC-category: the animals were divided into three groups according to their WBC count: WBC-decreased (leukopenia), WBC-normal (WBC count within the reference interval), WBC-increased (leukocytosis). The classification into these groups was made according to the reference intervals of Hengrave-Burri et al.^27^.
- Normoblasts-category: the animals were divided into two groups according to the occurrence of normoblasts in the peripheral blood: normoblasts-yes and normoblasts-no.

Only those animals for which all the aforementioned parameters were available were included in the study.

### Statistical analysis

#### Descriptive statistics

The WBC ratios were characterized using descriptive statistics in SAS (SAS Enterprise Guide 7.1) [mean (M), standard deviation (SD), median (Mdn), minimum (Min), maximum (Max), lower quartile (Q1), upper quartile (Q3)].

#### Generalized linear modeling

Modeling was conducted applying the software R^28^. The five leukocyte ratios (NLR, BLR, BNLR, BNR, and LMR) were examined as dependent variables using generalized linear models with Gaussian assumption for effects of several fixed factors in each two basic models. To achieve normality, NLR values were log transformed. Both models included species, sex, and age as important demographic factors as well as the month of sample collection (season). Model 1 also included BCS, WBC count, and PCV. Model 2 additionally included BCS-category, WBC-category, Anemia-category, and Normoblasts-category. Models were calculated using the “lme4” package^29^. An automated model selection approach was utilized to choose the most relevant combinations of fixed factors from the two basic models. The model selection process involved calculating all possible combinations of variables within each basic model using the “dredge()” function from the R package “MuMIn”^30^. The optimal combination of factors from a set of multiple candidate models was determined using the Akaike Information Criterion (AIC), following the methodology outlined by Burnham and Anderson^31^. To address small sample sizes, the corrected AIC (AICc) method recommended by Hurvich and Tsai^32^ was employed. Models with the highest statistical support and those with similarly low AICc values (∆AICc < 2) were considered for interpretation. Post hoc tests, specifically Tukey tests, were conducted to examine the significant effects of factors with more than two categories. These tests were performed using the “multcomp” package^33^. *P-* values less than 0.05 were considered as statistical significant.

## Results

**Fig. 1:**
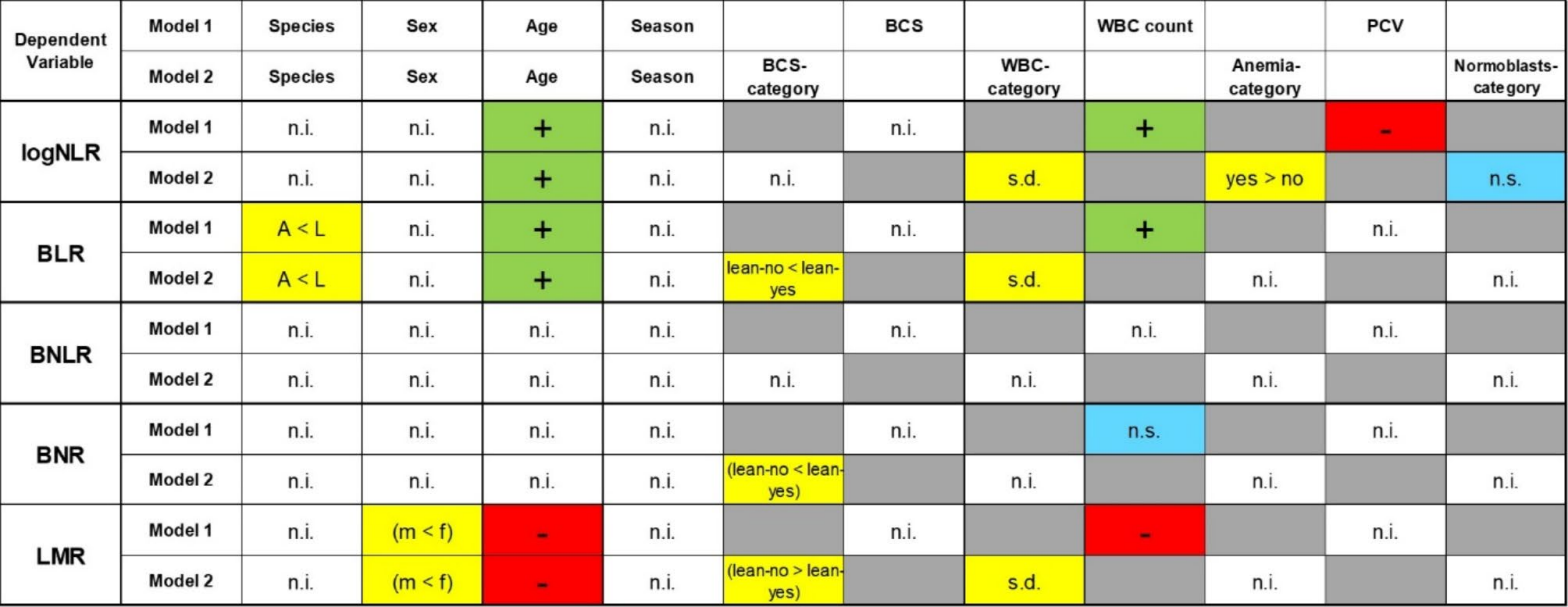
Simplified schematic presentation of the generalized linear model results of WBC ratios of 307 South American camelids (275 alpacas and 32 llamas) that were presented to a veterinary teaching hospital. Since some parameters are based on more than one single *p*-value, the presentation has been schematized and symbols have been used for better readability. The detailed statistical results can be found in Supplementary Table S2. NLR: neutrophil-to-lymphocyte ratio, BLR: band neutrophil-to-lymphocyte ratio, BNLR: band neutrophil-to-neutrophil-to-lymphocyte ratio, BNR: band neutrophil-to-neutrophil ratio, LMR: lymphocyte-to-monocyte ratio, A: alpaca, L: llama, m: male, f: female, n.i.: not included in the model, n.s.: not significant (*p* ≥ 0.05), s.d.: significant difference (*p* < 0.05), +: the WBC ratio increases with increasing independent variable, -: the WBC ratio decreases with increasing independent variable. Brackets indicate a statistical trend (0.1 > *p* > 0.05). The number of animals included in the LMR was only *n* = 282, as no LMR could be determined in the animals in which no monocytes were differentiated in the blood smear.

A detailed overview of the results of the descriptive statistics can be found in Supplementary Table S1. The results of the generalized linear models are displayed in Supplementary Table S2. Figure 1 provides a simplified schematic illustration of the modeling results, represented by the results of the models with lowest *AICc* values (best models). Relevant findings for each ratio will be explained in the following paragraphs.

### Neutrophil-to-lymphocyte ratio (NLR)

The median NLR of the investigated animals was 4.32 (Mdn; IQR: 2.31–7.81) and revealed a broad range with values up to 99. Significant effects on NLR were found for age, WBC count, WBC-category, Anemia-category, and PCV. Animals with leukocytosis had a higher NLR of 9.61 (Mdn; IQR: 6.45–18.8) than animals with a leukocyte count in the reference interval (Mdn: 3.84; IQR: 2.51–7.10) or animals with leukopenia (Mdn: 2.84; IQR: 1.76–5.07). Animals with anemia revealed a higher NLR (Mdn: 5.20; IQR: 2.69–9.03) than animals without anemia (Mdn: 4.09; IQR: 2.18–7.10). Increasing age as well as decreasing PCV led to increasing NLR. Although the occurrence of normoblast was included in the best model of Model2, modeling did not reveal any significant effects on NLR. Species, sex, season, BCS, and BCS-category were not included in the models with lowest AICc values and therefore probably had no particular influence on the NLR.

## Band neutrophil-to-lymphocyte ratio (BLR)

The BLR of the animals revealed a median of 0.24 (IQR: 0.07–0.87) and ranged up to 11. Species, age, BCS-category, WBC count, WBC-category led to significant differences in this parameter. Alpacas had lower BLR (Mdn: 0.23; IQR: 0.07–0.84) than llamas (Mdn: 0.28; IQR: 0.09–1.47), lean animals had higher BLRs (Mdn: 0.41; IQR: 0.12–1.15) than non-lean animals (Mdn: 0.15; IQR: 0.05–0.53). Animals with leukocytosis had higher BLRs (Mdn: 0.55; IQR: 0.20–1.76) than animals with a leukocyte count in the reference interval (Mdn: 0.19; IQR: 0.05–0.72) or animals with leukopenia (Mdn: 0.25; IQR: 0.05–0.79). Sex, season, BCS, PCV, Anemia-category, and Normoblasts-category were not included in the best models.

## Band neutrophil-to-neutrophil-to-lymphocyte ratio (BNLR)

The median BNLR was 3.66 × 10^–3^ (IQR: 1.17 × 10^–3^ − 14.20 × 10^–3^); this WBC ratio had a wide range of up to 185.19 × 10^–3^.

None of the investigated parameters were included in the best model.

### Band neutrophil-to-neutrophil ratio (BNR)

The BNR was 0.06 (Mdn; IQR: 0.02–0.15), with the highest value in a single animal of 0.87. Significant differences in the BNR were shown for the parameter BCS-category. Investigations of BCS-category revealed a statistical trend; lean animals had higher values (Mdn: 0.08; IQR: 0.02–0.23) than non-lean animals (Mdn: 0.04; IQR: 0.01–0.11). Although the WBC count was included in the best model of Model 1, no significant effect on BNR was detected. The parameters species, sex, age, season, BCS, WBC-category, Anemia-category, PCV, and Normoblasts-category were not included in the best models.

### Lymphocyte-to-monocyte ratio (LMR)

The LMR had a median of 7 (IQR: 3.54–14.67) and a maximum of 97. As there can be no 0 values in the LMR, there is always a lower limit above 0. The lowest value recorded in an animal was 0.13. Significant influences were observed for the parameters age, WBC count, and WBC-category. The investigation of sex and BCS-category revealed statistical trends. LMR decreased with age of the animals. Leukocytosis was associated with lower LMR (Mdn: 4; IQR: 1.8–7.33), whereas animals with a leukocyte count in the reference interval (Mdn: 8; IQR: 4–17) or animals with leukopenia (Mdn: 8.25; IQR: 3.67-15) revealed higher LMR. Although there was a statistical trend concerning sex, both males and females revealed a median of 7 in the LMR. However, the mean in females (12.51) was slightly higher than in males (10.25). Furthermore, lean animals had lower ratios (Mdn: 5.71; IQR: 3.11–12.63) than non-lean animals (Mdn: 8.2; IQR: 4–15).

## Discussion

In our study, we presented data on different WBC ratios from a large number of hospitalized SAC in relation to species, sex, age, season, BCS, WBC count, and PCV for the first time. The NLR, BLR, and LMR were found to be significantly influenced by the age of the animal and the WBC count. Furthermore, an association of individual WBC ratios with species, nutritional status, or an anemic condition could be detected. Although the median age of the llamas in the population we studied was higher than that of the alpacas, this difference turned not out to be statistically significant (*p* = 0.15; Mann-Whitney U test), we therefore assume that this did not bias the findings concerning species and age. The influence of species, sex, age, and season on neutrophils and lymphocytes in SAC has been previously described^27,34^. Hengrave Burri and colleagues found higher numbers of each of the segmented and band neutrophils as well as lymphocytes in llamas than alpacas. They found no difference between male and female animals in either species, but reported that crias of both species had significantly more lymphocytes than adult animals^27^. However, Husakova and colleagues found lower numbers of both lymphocytes and neutrophils in alpaca crias than in adult alpacas^34^. They also found lower lymphocyte counts in males than in females and lower numbers of neutrophils and monocytes in winter than in summer^34^. In our evaluation, we did not find any influence of the month of sampling on any of the WBC ratios. The findings concerning lymphocytes in the study by Husakova et al.^34^ is reflected in a trend to lower LMR in males than females in our population. The fact that NLR increases with age of the animal was also observed in another population where a retrospective examination of hematologic findings in healthy female alpacas from an experimental herd showed a moderate correlation between NLR and age (r_s_=0.59; *p* < 0.001)^35^. An increase in neutrophils could perhaps be a compensation for a decrease in neutrophil activity with age. Wenisch and colleagues found a reduced phagocytic ability and bactericidal activity of neutrophils in elderly humans^36^. However, the extent to which this can be applied to SAC remains unclear. A higher BLR in llamas in this study compared to alpacas is consistent with the findings of Hengrave Burri et al.^27^ who found that band neutrophils were significantly higher in llamas than in alpacas. The observation by Husakova et al.^34^ that female alpacas had higher lymphocyte counts than males is also reflected in the slightly higher LMR of females in our study. The aforementioned associations of BCS with neutrophils and lymphocytes^2^ are also reflected in the NLR, BLR, and LMR, although it should be noted that part of the population examined in the present study was already included in the previous study^2^.

Already in 1983 Montes et al. specified the NLR for 30 Chilean alpacas of both sexes as 0.73 ± 0.26 with a range of 0.2–1.1^9^. Hajduk examined 29 alpacas of both sexes that were imported to Australia and gave a reference interval for the NLR of 0.5–2.9 in 1992^7^. The NLR of 89 adult llamas was given as 1.54 without further specification whether this value represented the mean or median by Fowler as well as Foster et al.^12,37^. When compared with the NLRs obtained in the present study, it is noticeable that those previously reported NLRs were lower, which may be explained by the fact that those values were obtained from healthy animals, whereas the ratios of the present study were obtained from hospitalized animals. In a study of systemic inflammation in alpacas, six healthy castrated animals were treated with LPS and compared with a control group of another six healthy castrated individuals at various time points^8^. The NLRs of the control group were higher than those previously reported values and similar to our results, ranging from 3.06 to 4.62, while the NLRs of the LPS group increased to 9.61 after 12 h and reached a maximum of 13.67 after 24 h. Those findings by Passler et al.^8^ illustrate that NLR is a suitable indicator for inflammation in alpacas. However, when interpreting the NLR, it is important to keep in mind that NLR is not a specific parameter. In addition to inflammation, stress, for example transport stress, can lead to an increase in neutrophils and a decrease in lymphocytes^38^, which also results in an increased NLR, which has been shown in other species like cattle^39^ or dogs^13^.

The NLR is mentioned in other papers on hematology in SAC, but without giving specific values^10,11,40,41^. According to Fowler and Zinkl^10^ and van Houten et al.^11^, the NLR in SAC is similar to that of humans, dogs, cats, and horses. Nonetheless, it should be noted that stress-induced neutrophilia can be particularly pronounced in SAC, and according to Bildfell et al.^40^ there can also be a significant stress-induced increase in band neutrophils. This potential for high NLR is also evident in our data: a quarter of the animals in our study had an NLR > 7.81. According to Zahorec^42^, in a graduation for NLR in humans, values above 7 indicate inflammation or stress. Whether this classification is also suitable for SAC should be investigated in further studies on NLR. Hickman reported that NLR was significantly higher in mice with chronic stress compared to mice without stress^14^. The NLR was further reported to be a better parameter to detect chronic stress than measurement of cortisol^15^. If this also applies to SAC, the NLR would be an easy and inexpensive parameter to measure or indicate stress. Furthermore, there may be an association between NLR and the severity of gastric ulcers; data from human medicine showed that patients with peptic ulcer perforation had significantly higher NLR than both patients with peptic ulcer disease and patients in the control group^43^.

The age-related decline observed for LMR was also observed in the healthy population of female alpacas mentioned above with a weak association between age and LMR (r_s_=-0.31; *p* < 0.01)^35^. To the best of our knowledge, no other study on LMR or BLR, BNLR or BNR in SAC can currently be found in the literature. In both human^44,45^ and veterinary medicine^21^, LMR serves as a prognostic marker for various tumor diseases, and could therefore also be useful for SAC. Since monocytosis can also be part of a stress leukogram in some species^46^, a decrease in LMR could possibly indicate stress in addition to an increase in NLR. When interpreting the LMR, it must be considered that only a few monocytes are detected in the blood smear when evaluating 200 WBCs, and one misclassified cell can result in a large difference.

BNLR and BLR were investigated in canine inflammatory response syndrome by Pierini et al.^24^. In contrast to NLR, which was significantly lower in septic dogs than in aseptic dogs, no associations were found for BNLR and BLR. However, Gori et al. found significantly higher values for BLR and BNLR in cats with feline systemic inflammatory response syndrome and in cats with sepsis than in healthy cats^23^. The significance of these BNLR, BLR, and BNR for SAC should be investigated in further studies.

### Limitations

As this was a retrospective study on hospitalized animals, the given ratios are not intended to serve as reference values, but only for orientation. It should be noted that the animals presented to the clinic suffered from one or more pathological conditions, and therefore they do not reflect the condition of healthy animals. As the evaluation is based on a large data set over several years, including a large proportion of SAC presented to our clinic, we believe that the results reflect a representative overview of the conditions in diseased SAC. The diagnoses of the animals were not included in the evaluation, as the available data would not be sufficient to compare different diagnoses. Implementing single diagnoses in the evaluation would further have resulted in too many variables, so we focused on alterations in the standardized hematologic parameters and BCS. The BCS in SAC by palpation of the lumbar spine as a clinical parameter was shown to give an objective estimation of the nutritional status, even when it is assessed by different examiners^47^. Common reasons for presentation of SAC in our clinic are nonspecific weakness and anorexia, acute abdominal pain, dyspnea and diarrhea. A detailed description of the type and frequency of pathological findings in the SAC presented in our clinic was recently published elsewhere by Neubert and colleagues^48^. The most frequent changes were pathological alterations of the gastrointestinal tract (48.7%), the liver (31.2%) and the respiratory tract (20.8%)^48^. However it should be noted that the incidence of individual diseases varies according to age and sex^48^, and a bias in these parameters cannot be ruled out on the basis of our data. For a few of the animals admitted to the clinic, the exact date of birth was unknown and was therefore estimated. The age in days was an approximation for these animals. No differences between intact and neutered were investigated in the male animals. As the platelets had not been checked manually and were not included in the data set, no statement could be made about the PLR in this study.

### Conclusion and outlook

The results of this study show that the WBC ratios in hospitalized SAC can have wide ranges. Based on our results, it can be assumed that the WBC ratios vary depending on the species, sex, age, BCS, WBC count, and PCV of the animals. In analogy to other species, increased NLR and decreased LMR could be an indicator of stress in SAC and, like BNLR, BLR, and BNR, could be used as prognostic parameters for various diseases.

The fact that WBC ratios can be very easily determined from an existing WBC differential at no additional cost means that there is great potential for their use as diagnostic markers in everyday veterinary practice. Further studies are needed to investigate the impact of NLR on different disease patterns in SAC.

## Electronic supplementary material

Below is the link to the electronic supplementary material.

Supplementary Material 1

## Data Availability

The original contributions presented in the study are included in the article/supplementary material. Further inquiries can be directed to the corresponding author/s.

## Abbreviations

A: Alpaca
AIC: Akaike Information Criterion
BCS: Body Condition Score
BLR: Band neutrophil-to-lymphocyte ratio
BNLR: Band neutrophil-to-neutrophil-to-lymphocyte ratio
BNR: Band neutrophil-to-neutrophil ratio
f: Female
IQR: Interquartile range
L: Llama
LMR: Lymphocyte-to-monocyte ratio
m: Male
Max: Maximum
Mdn: Median
Min: Minimum
n.i.: Not included
NLR: Neutrophil-to-lymphocyte ratio
n.s.: Not significant
PCV: Packed cell volume
PLR: Platelet-to-lymphocyte ratio
Q1: Lower quartile
Q3: Upper quartile
s.d.: significant difference
SAC: South American camelid
SD: Standard deviation
WBC: White Blood Cell
